# Effect of Scanning Origin Location on Data Accuracy of Abutment Teeth Region in Digital Impression Acquired Using Intraoral Scanner for Removable Partial Denture: A Preliminary In Vitro Study

**DOI:** 10.3390/jcm11247392

**Published:** 2022-12-13

**Authors:** Eung-Yeol Kim, Junichiro Wada, Kazuki Sakamoto, Yurika Ishioka, Yuki Arai, Natsuko Murakami, Toshiki Yamazaki, Hironari Hayama, Miona Utsumi, Shusuke Inukai, Noriyuki Wakabayashi

**Affiliations:** 1 Department of Advanced Prosthodontics, Tokyo Medical and Dental University—TMDU, 1-5-45, Yushima, Bunkyo-ku, Tokyo 113-8510, Japan; 2 Department of Biomaterials Science, Turku Clinical Biomaterials Centre—TCBC, Institute of Dentistry, University of Turku, Itäinen Pitkäkatu 4B, 20520 Turku, Finland; 3 Department of Orthodontics, University of Texas Health Science Center at Houston School of Dentistry, 7500 Cambridge St., Houston, TX 77054, USA

**Keywords:** data accuracy, digital impression, intraoral scanner, linear accuracy, precision, removable partial denture, scanning origin, trueness

## Abstract

The aim of this study was to investigate the effect of scanning origin location on the data accuracy of removable partial denture (RPD) abutment teeth region in digital impressions acquired by an intraoral scanner. A mandibular partially edentulous model including the following target abutment teeth was used: the left second molar (#37); left first premolar (#34); and right second premolar (#45). The following scanning strategies were tested: the strategy starting from #37 to mesial direction (37M); strategies starting from #34 to mesial (34M) and distal directions (34D), and strategies starting from #45 to mesial (45M) and distal directions (45D). The evaluated measures were trueness, precision, and linear accuracy. One-way and two-way ANOVA were performed for the comparison of trueness and linear accuracy, while Kruskal–Wallis test was performed for the precision comparison (α = 0.05). 45M and 45D showed significantly superior trueness of #34 to 37M and 34D. 45M also showed significantly superior trueness of #45 to 34. 45D showed significantly inferior linear accuracy of #34 and superior linear accuracy of #45 compared to other strategies. It was concluded that scanning origin location would have an impact on data accuracy of RPD abutment teeth region in digital impressions acquired by intraoral scanner.

## 1. Introduction

Recently, digital impression-taking by an intraoral scanner (IOS) has been clinically applied for the fabrication of removable partial dentures (RPDs) more widely than ever [1,2,3]. Meanwhile, the applications of RPD fabrication directly from digital impressions by IOS are limited for the cases with a short-span mucosal area with remaining teeth both anterior and posterior to it [1,2]. For the cases with mucosal areas which are long-span and/or located posterior to the remaining dental arch, a functional impression using an occlusal rim or a wax denture with a framework is necessary to finalize the digital impression as the definitive impression [4]. Under such circumstances, previous studies have focused especially on the data accuracy of mucosal areas in digital impressions [5,6,7]. However, for partially edentulous dental arches with a variety of numbers and locations of mucosal areas and remaining teeth, it is still unclear how to improve the data accuracy of remaining teeth regions including RPD abutment teeth in digital impressions.

The data accuracy of remaining teeth regions in digital impressions can critically affect the fitting of RPD frameworks. The fitting of frameworks should be accurate to keep RPDs passive in the oral cavities to protect both the remaining teeth and denture-bearing mucosa [8]. Previous studies have indicated that the numbers and locations of the mucosal area [9], the size of scanner head [5], and the scanning strategy [10] would affect the full-arch data accuracy of digital impressions for partial edentulous dental arches. In previous studies, trueness, precision, and linear accuracy based on the distance between two representative points in digital data have been assessed to evaluate data accuracy of digital impression. According to the International Organization for Standardization (ISO, Geneva, Switzerland), trueness is defined as the deviation between the reference and target objects, and precision is defined as the variability between repeated measurements in the impression processes (ISO5725-1) [11]. In previous studies, the following findings have been reported: (1) digital impression by IOS would reveal higher trueness and lower precision of both mucosal area and whole dental arch than conventional impression using elastic impression materials [5]; (2) a lack of landmark would deteriorate linear accuracy including mucosal area [12], and (3) a long-span mucosal area and/or multiple mucosal areas would deteriorate the precision of whole dental arch in digital impression [9]. Although those studies did not focus solely on the data accuracy of remaining teeth regions, it has been indicated that the above-mentioned factors could somehow affect the data accuracy of remaining teeth regions. However, no study has investigated how to improve the data accuracy of digital impressions acquired by IOS focusing on RPD abutment teeth regions.

In digital impression-taking by IOS, the scanning strategy specified by manufacturers is initiated at the most posterior region, followed by the scanning of occlusal surfaces. Subsequently, the lingual/palatal surfaces are scanned and finalized with the scanning of the buccal/labial surfaces [13]. On the other hand, previous studies have indicated that the longer distance from the scanning origin would deteriorate data accuracy both in sound dentition [14] and partially edentulous dental arch [15]. Additionally, it has been reported that the lack of landmarks in the mucosal area would deteriorate the data accuracy of digital impressions for partially edentulous dental arches [6,7]. Therefore, the location of scanning origin might critically affect the data accuracy of digital impressions for partially edentulous dental arches. Nevertheless, for the partially edentulous dental arches, no study has evaluated the effect of scanning origin location on the data accuracy of digital impressions, while the effect of scanning route on the data accuracy of digital impressions has been previously investigated [10].

In this study, several digital impressions for a mandibular partially edentulous dental arch model were taken by an IOS with five different scanning strategies including several locations of the scanning origin. As measures of data accuracy, the trueness, precision, and linear accuracy were evaluated. This study aimed to investigate the effect of scanning origin location on the data accuracy of remaining teeth regions in digital impressions acquired by an IOS. The tested null hypothesis was “the scanning origin location would not affect the data accuracy of remaining teeth regions in digital impressions acquired by an IOS”.

## 2. Materials and Methods

### 2.1. Reference Model

A mandibular Kennedy class II, modification 1 partially edentulous dental arch model (E50-528; Nissin, Tokyo, Japan) was used as the reference model in this study. In the reference model, the following four teeth were missing: the left second premolar (#35); left first molar (#36); right first molar (#46), and right second molar (#47). The target teeth to be evaluated were the following three teeth, supposing the RPD abutment teeth: the left first premolar (#34), left second molar (#37), and right second premolar (#45). The artificial teeth of reference model, including the target teeth, were made of melamine resin, while its residual ridge was made of epoxy resin. To calculate the linear accuracy, a stainless-steel spherule with a diameter of 3 mm was placed on the buccal cusp of each target tooth (the mesial buccal cusp for molars) and lingual frenum.

### 2.2. Digital Data Acquired Using a High-Accuracy Industrial Scanner (Reference Data)

For evaluating the data accuracy of digital impression using an IOS, the reference data were acquired using a high-accuracy industrial scanner (ATOS TripleScan 16M; GOM, Braunschweig, Germany) (*n* = 1). In accordance with the previous study, this scanner shows trueness of 3 µm for objects with a jaw size [16]. Using 3D-modeling software (Geomagic Studio 2014; 3D Systems, Rock Hill, SC, USA), the acquired data were converted to standard triangulated language (STL) files, followed by the trimming at the gingivobuccal fold and the deepest lingual point. The preliminary experiment was performed to confirm the errors caused by trimming, showing that the average value of the errors was 1.1 μm (*n* = 10). The trimmed STL files were used as the reference data.

### 2.3. Scanning Strategies for Digital Data Acquisition Using an Intraoral Scanner (IOS Data)

**Figure 1:**
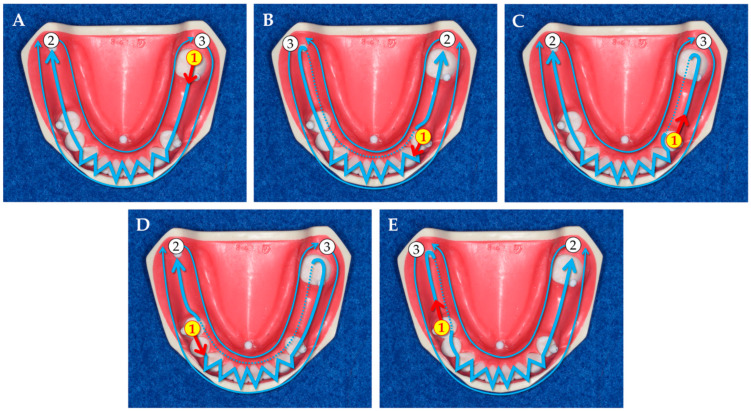
Scanning strategies used in this study. Each strategy basically starts with a route on the occlusal surface (1), followed by a route on the lingual surface (2), and then on the buccal surface (3). Based on the location of the scanning origin and scanning direction from the scanning origin, scanning strategies were defined as follows: (**A**) scanning strategy initiated at #37 and headed to the mesial direction (specified by the manufacturer): 37M; (**B**) scanning strategy initiated at #34 and headed to the mesial direction: 34M; (**C**) scanning strategy initiated at #34 and headed to the distal direction: 34D; (**D**) scanning strategy initiated at #45 and headed to the mesial direction: 45M; and (**E**) scanning strategy initiated at #45 and headed to the distal direction: 45D.

The digital impressions of the reference model were acquired by full-arch scanning using an IOS (TRIOS3; 3Shape, Copenhagen, Denmark) to be evaluated. The five different scanning strategies performed in this study were defined based on the location of scanning origin and scanning direction as follows: scanning strategies (1) initiated at #37 and headed to the mesial direction (37M) (specified by the manufacturer [13]); (2) initiated at #34 and headed to the mesial direction (34M); (3) initiated at #34 and headed to the distal direction (34D); (4) initiated at #45 and headed to the mesial direction (45M); and (5) initiated at #45 and headed to the distal direction (45D) (Figure 1). One experienced operator (E.-Y.K.) performed all scanning. For each scanning strategy, 10 digital impression data were acquired and trimmed in the same manner as the acquisition of reference data, followed by conversion to the STL files as the IOS data (*n* = 10/scanning strategy). The sample size was determined according to the previous study [15].

### 2.4. General Trueness (T_G_), Local Trueness (T_L_), and Precision

**Figure 2:**
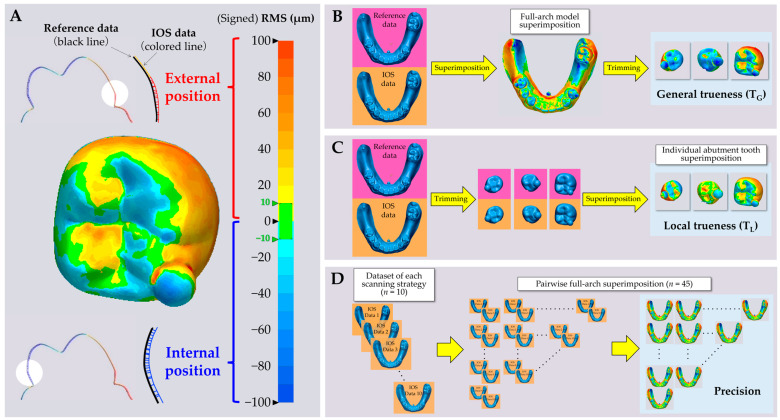
Color map image structured based on signed RMS value on and workflows of the evaluation of trueness and precision. (**A**): Positive scales in color map represent that IOS data are positioned at an external area, while negative scales represent that IOS data are positioned at an internal area relative to the reference data; (**B**): workflow of evaluation of the general trueness based on full-arch model superimposition (T_G_); (**C**): workflow of evaluation of the local trueness based on individual abutment tooth superimposition (T_L_); and (**D**): workflow of evaluation of the precision for full-arch IOS data.

To calculate the trueness and precision, the root mean square (RMS) value (µm) of deviation between two data was calculated using the following equation:

$$\mathrm{RMS}=\sqrt{\frac{\sum_{i=1}^{n}{\left(d_{e,i}-d_{r,i}\right)}^{2}}{n}}$$

where *n* is the total number of STL structural points of evaluated data; *d_e,i_* is the value of measurement of *i*th structural point on evaluated data, and *d_r,i_* is the value of measurement of *i*th structural point on reference data. The results of RMS calculations are shown as a color map image with the allocation of plus or minus signs based on the position of structural point on evaluated data relative to reference data, indicating that a positive RMS represents an externally positioned structural point on the evaluated data, while a negative RMS represents an internally positioned structural point on the evaluated data (Figure 2A).

To evaluate the general trueness based on full-arch model superimposition (T_G_), IOS data for full-arch model was superimposed on the reference data using best-fit algorithm of 3D analysis software (Geomagic Control X; 3D Systems, Rock Hill, SC, USA) [17]. Then, the RMS value at only individual target abutment tooth region of superimposed IOS data was calculated, representing T_G_ for each target abutment tooth (Figure 2B). Meanwhile, to evaluate the local trueness based on individual abutment tooth superimposition (T_L_), both the reference and IOS data were trimmed at each abutment tooth region beforehand, followed by data superimposition using best-fit algorithm. Then, the RMS value was calculated, representing T_L_ for each target abutment tooth (Figure 2C). In the trueness evaluation, a higher RMS value indicated inferior trueness.

In addition, the precision for the full-arch model in IOS data was compared among the five scanning strategies. The pairwise superimposition among the dataset of each scanning strategy (*n* = 10) by selecting all pairs from the 10 data was performed using best-fit algorithm. Then, the RMS value among each pair was calculated representing precision (*n* = 45/scanning strategy) (Figure 2D). In the precision evaluation, a higher RMS value indicated inferior precision.

### 2.5. Linear Accuracy

**Figure 3:**
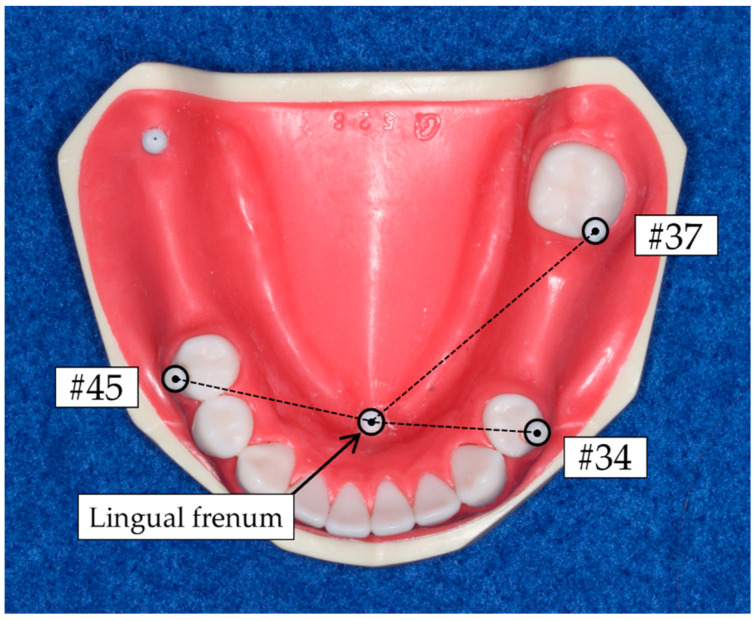
Distance between the central coordinate of the stainless-steel spherule placed on the lingual frenum and that on the buccal cusp of each target tooth was evaluated for the linear accuracy analysis.

To evaluate the linear accuracy, the distance between central coordinate of stainless-steel spherule placed on the lingual frenum and that on the buccal cusp of each target tooth was calculated both in reference and IOD data (Figure 3). The change ratio in the distance based on reference data was evaluated for each abutment tooth in IOS data, representing the linear accuracy. The linear accuracy was calculated using following equation:

$$A_{L}=\frac{D_{I}-D_{R}}{D_{R}}\times 100$$

where *A_L_* is the linear accuracy (%); *D_I_* is the distance from lingual frenum in IOS data (mm), and *D_R_* is the distance from lingual frenum in reference data (mm). A positive value of the linear accuracy indicated the distance between lingual frenum and each abutment tooth in IOS data was greater than that in reference data. Regarding the absolute value of linear accuracy, a higher value indicated inferior accuracy.

### 2.6. Statistical Analysis

For each abutment tooth, the trueness (T_G_ and T_L_), precision, and linear accuracy were statistically compared among five scanning strategies. Shapiro–Wilk test indicated that all measures showed the normality of data, while Levene’s test indicated that all the measures except for precision showed the equality of variance of data. Therefore, a one-way ANOVA with a Tukey multiple comparison post hoc test was performed for the comparisons of trueness (T_G_ and T_L_) and linear accuracy. Additionally, a two-way ANOVA based on the scanning strategy and target abutment tooth for their comparison. On the other hand, a Kruskal–Wallis test followed by pairwise comparisons with Bonferroni correction was performed for the comparison of precision. All statistical analyses were performed using statistical software (SPSS Statistics v28.0, IBM, Redmond, WA, USA) with a significance level set at 0.05.

## 3. Results

**Figure 4:**
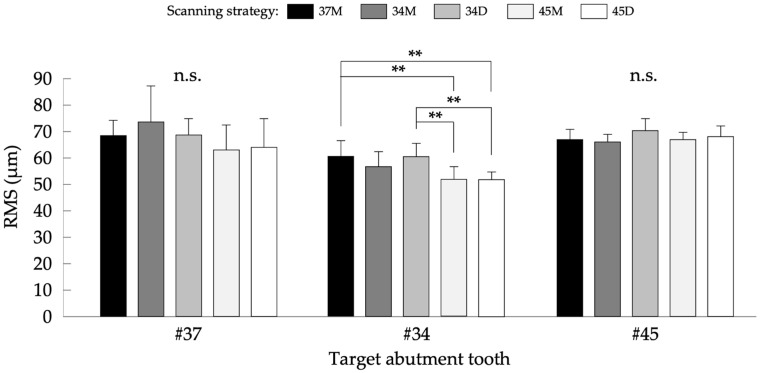
Results of the comparisons of T_G_ for each target abutment tooth among five scanning strategies. n.s.: not significant; and **: *p* < 0.01.

**Figure 5:**
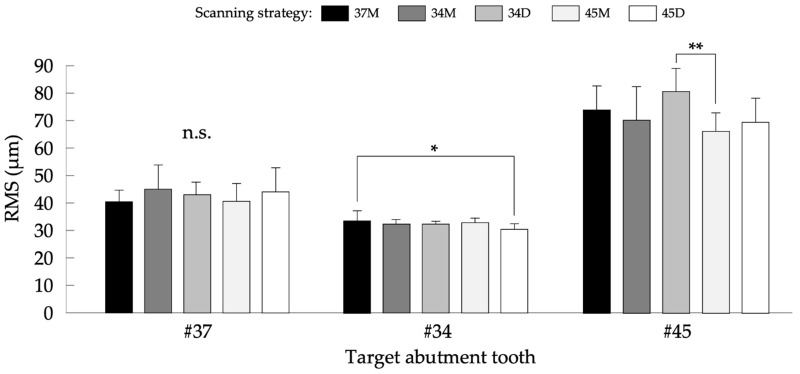
Results of the comparisons of T_L_ for each target abutment tooth among five scanning strategies. n.s.: not significant; *: *p* < 0.05; and **: *p* < 0.01.

**Figure 6:**
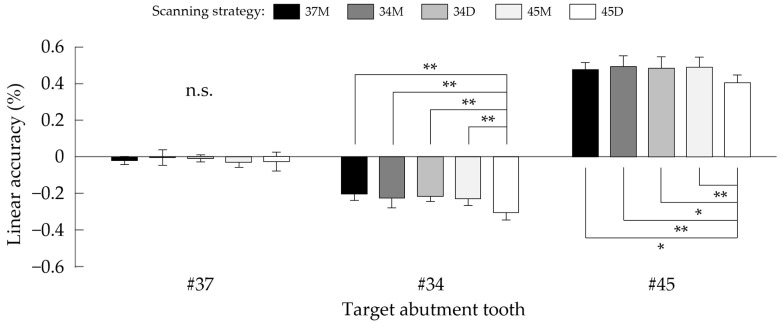
Results of the comparison of linear accuracy for each target abutment tooth among five scanning strategies. n.s.: not significant; *: *p* < 0.05; and **: *p* < 0.01.

Table 1 shows the *p*-values acquired by a two-way ANOVA and reveals that both the scanning strategy and target abutment tooth significantly affected T_G_, T_L_, and linear accuracy. On the other hand, Figure 4, Figure 5 and Figure 6 show the results of comparisons acquired by a one-way ANOVA and a Tukey multiple comparison post hoc test.

Figure 4 shows the results of comparisons of T_G_ among five scanning strategies. The mean values ± standard deviations (SD) (µm) in #37 were 69.1 ± 5.7, 74.5 ± 13.5, 69.4 ± 6.1, 63.7 ± 9.4, and 64.7 ± 10.9 in IOS data acquired with 37M, 34M, 34D, 45M, and 45D strategies, respectively. One-way ANOVA showed no significant difference among the scanning strategies (*p* = 0.109). The mean values ± SD (µm) in #34 were 61.3 ± 5.8, 57.4 ± 5.7, 61.1 ± 5.1, 52.5 ± 4.9, and 52.4 ± 3.0 with 37M, 34M, 34D, 45M, and 45D strategies, respectively. One-way ANOVA showed a significant difference among the scanning strategies (*p* < 0.001), and 37M and 34D revealed significantly higher T_G_ than 45M (*p* = 0.002 and 0.003 for 37M and 34D, respectively) and 45D (*p* = 0.002 for both 37M and 34D). The mean values ± SD (µm) in #45 were 67.5 ± 4.1, 66.9 ± 2.8, 71.3 ± 4.2, 67.7 ± 2.8, and 68.9 ± 3.9 with 37M, 34M, 34D, 45M, and 45D strategies, respectively. One-way ANOVA showed no significant difference among the scanning strategies (*p* = 0.070).

Figure 5 shows the results of the comparisons of T_L_ among five scanning strategies. The mean values ± SD (µm) in #37 were 40.6 ± 3.9, 45.1 ± 8.6, 42.9 ± 4.5, 40.6 ± 6.4, and 44.1 ± 8.6 with 37M, 34M, 34D, 45M, and 45D strategies, respectively. One-way ANOVA showed no significant difference among the scanning strategies (*p* = 0.473). The mean values ± SD (µm) in #34 were 33.7 ± 3.6, 32.4 ± 1.6, 32.5 ± 1.1, 33.1 ± 1.3, and 30.6 ± 1.9 with 37M, 34M, 34D, 45M, and 45D strategies, respectively. One-way ANOVA showed a significant difference among the scanning strategies (*p* = 0.024), and 37M revealed significantly higher T_L_ than 45D (*p* = 0.015). The mean values ± SD (µm) in #45 were 74.3 ± 8.6, 70.6 ± 12.0, 81.0 ± 8.3, 66.5 ± 6.6, and 69.7 ± 8.7 with 37M, 34M, 34D, 45M, and 45D strategies, respectively. One-way ANOVA showed a significant difference among the scanning strategies (*p* = 0.010), and 34D revealed significantly higher T_L_ than 45M (*p* = 0.007).

Figure 6 shows the results of the comparisons of linear accuracy (change ratio in distance from lingual frenum) among five scanning strategies. The mean values ± SD (%) in #37 were −0.019 ± 0.022, −0.004 ± 0.043, −0.011 ± 0.020, −0.030 ± 0.028, and −0.027 ± 0.052 with 37M, 34M, 34D, 45M, and 45D strategies, respectively. One-way ANOVA showed no significant difference among the scanning strategies (*p* = 0.431). The mean values ± SD (%) in #34 were −0.203 ± 0.035, −0.224 ± 0.056, −0.213 ± 0.029, −0.230 ± 0.035, and −0.305 ± 0.038 with 37M, 34M, 34D, 45M, and 45D strategies, respectively. One-way ANOVA showed a significant difference among the scanning strategies (*p* < 0.001), and 45D revealed a significantly lower change ratio than 37M (*p* < 0.001), 34M (*p* < 0.001), 34D (*p* < 0.001), and 45M (*p* = 0.001). The mean values ± SD (%) in #45 were 0.475 ± 0.039, 0.493 ± 0.059, 0.482 ± 0.064, 0.490 ± 0.056, and 0.405 ± 0.041 with 37M, 34M, 34D, 45M, and 45D strategies, respectively. One-way ANOVA showed a significant difference among the scanning strategies (*p* = 0.003), and 45D revealed a significantly lower change ratio than 37M (*p* = 0.034), 34M (*p* = 0.005), 34D (*p* = 0.016), and 45M (*p* = 0.006).

**Figure 7:**
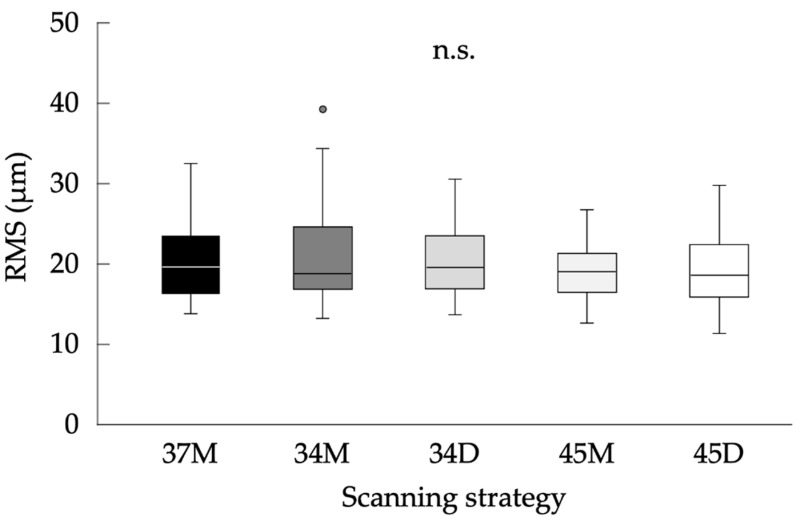
Results of the comparison of precision for full-arch model among five scanning strategies. The gray dot above the box plot of 34M indicates the outlier. n.s.: not significant.

Additionally, Figure 7 shows the results of the comparison of precision for full-arch model among five scanning strategies. The median values (interquartile ranges: IQR) (µm) were 19.5 (6.7), 18.8 (7.2), 19.5 (6.2), 19.0 (4.6), and 18.6 (6.3) within IOS datasets acquired with 37M, 34M, 34D, 45M, and 45D strategies, respectively. Kruskal–Wallis test showed no significant difference among the scanning strategies (*p* = 0.542).

## 4. Discussion

In this study, digital impressions of the mandibular partially edentulous dental arch model were taken by an IOS using five scanning strategies including three different scanning origins (#37, #34, and #45), followed by the comparisons of data accuracy (trueness, precision, and linear accuracy) among scanning strategies. The overall results revealed that the location of scanning origin played a significant role in data accuracy, and scanning direction as well. Therefore, the null hypothesis was rejected.

Regarding trueness evaluation, the general trueness based on full-arch model superimposition (T_G_) and local trueness based on individual abutment tooth superimposition (T_L_) were evaluated. T_G_ was the RMS value calculated with data trimming at each target abutment tooth region after superimposition of the reference and IOS data, representing trueness mainly affected by locational condition of abutment tooth in whole dental arch. Previous studies indicated that tooth located at posterior region would show inferior data accuracy [18,19]. Although this study focused on the comparison among scanning strategies, #37 tended to show inferior T_G_ compared to #34 and #45 (Figure 4). Meanwhile, the effect of locational condition of each abutment tooth might be demagnified by full-arch superimposition using best-fit algorithm. Thus, the RMS value calculated with superimposition of reference and IOS data after data trimming at each target abutment tooth region (T_L_) was also evaluated, representing trueness mainly affected by local error in geometry. The results of this study revealed that scanning strategies initiated at #45 (45M and 45D) showed superior trueness both in T_G_ and T_L_. This finding suggested that the scanning strategy specified by manufacturers would be not always recommended to achieve accurate digital impression data using an IOS. Nevertheless, it was also suggested that the scanning direction from scanning origin would not have an impact on the trueness of digital impression for partially edentulous dental arch. In this study, the highest RMS values in T_G_ and T_L_ were 74.5 µm (Figure 4) and 81.0 µm (Figure 5), respectively. Previous studies have reported that the trueness of the conventional impression using elastic impression material ranged from 122 to 157 µm [5] and fitting accuracy of RPD frameworks fabricated from conventional impression was 114 µm [20]. Therefore, from the viewpoint of trueness, data accuracy of digital impression for partially edentulous dental arch would be equal or superior to that of conventional impression, suggesting that data accuracy of digital impression would be acceptable to fabricate RPD frameworks with appropriate fitting accuracy.

Regardless of scanning strategy, the precision of acquired digital impression data was about 20 µm which is dramatically superior to previously reported precision of conventional impression (77–119 µm) [5]. Additionally, there was no significant difference among five scanning strategies (Figure 6). Therefore, it was indicated that precision of digital impression for partially edentulous dental arch would not be affected by location of scanning origin and scanning direction and would be sufficient for clinical application.

In the linear accuracy of #34, 45D showed most inferior accuracy (Figure 7). This finding differed from the tendency found in trueness evaluation, indicating that linear accuracy would be affected by both scanning origin and direction. In previous studies, it was suggested that the data accuracy of mucosal area would be deteriorated due to lack of a landmark for data stitching [6,7,12]. In the scanning strategies with scanning direction directly from scanning origin to mucosal area, errors of data stitching at initial phase of scanning might cause an undesirable effect on data accuracy of whole dental arch. Conversely, 45D showed most superior linear accuracy in #45, suggesting that the data accuracy around scanning origin would not be affected by scanning direction. However, it is still unclear how mucosal area facing scanning origin would affect data accuracy. Conversely to previous studies that revealed buccally displacement of tooth at posterior region [21], the change ratios found in #37 were small regardless of scanning strategies. It was also reported that remaining teeth posterior to mucosal area would be displaced to mesial direction [15], suggesting that the mucosal area anterior to #37 could demagnify the change ratios in #37. Nevertheless, the most inferior linear accuracy found in this study was 0.49% (Figure 6), while the American Dental Association (ADA) accepts linear dimensional change of less than 0.5% at 24 h after impression-taking using conventional material [22]. Therefore, the linear accuracy of digital impression for partially edentulous dental arch would be acceptable for clinical application, especially for fabrication of RPD framework.

Previous study reported that the long distance from scanning origin would deteriorate data accuracy [14], suggesting that it would not be always better to locate the scanning origin at most posterior region of dental arch as manufacturers recommend. However, in several previous studies, the scanning strategies initiated at incisors or premolars would not be necessarily superior to other scanning strategies including manufacturer-specified strategy in sound dental arches [23,24]. In this study, the scanning strategies initiated at #34 which was relatively middle region of remaining dental arch (34M and 34D) did not show superior data accuracy overall compared to #45M. This finding suggested that the scanning origin located at end of remaining dental arch but not at the remaining tooth isolated from other remaining teeth via the mucosal area would be recommended to acquire superior accuracy of RPD abutment teeth in digital impressions using an IOS. Additionally, it was also suggested that the scanning strategies directed from scanning origin directly to mucosal area would not be recommended.

This study had several limitations. First, a Kennedy class II, modification 1 mandibular model was the only model evaluated. Additionally, this study was conducted as an in vitro study using a resin model. Therefore, it is unclear whether our findings can be generalized for actual oral cavities with partially edentulous dental arches on the maxillary jaw and/or those with other Kennedy classifications. Second, we evaluated only RPD abutment teeth regions except for precision evaluation. Further studies are necessary to investigate how the scanning origin affects data accuracy of mucosal area and remaining teeth other than RPD abutment teeth. Third, there was the potential for a variation in number of images captured during scanning among evaluated five scanning strategies. A previous study indicated that number of images would affect data accuracy of digital impressions acquired using IOS [25]. Although we carefully tried to maintain the number of images from 2600 to 2800 for each scanning regardless of scanning strategies, strategy with the shortest scanning route (37M) might capture a small number of images compared to other strategies. Therefore, the results would be potentially affected by variation of captured images. Fourth, only one operator who are well-trained performed all scanning to eliminate the data variability. However, it was reported that operator’s experience in using IOS affected the accuracy of digital impression [26], indicating that this study would potentially risk the reliability of data. Fifth, a single IOS was used in this study. Several studies have revealed that the scanner type could play a role in data accuracy of digital impressions [3,9,10,13,14,19], suggesting that it is still unclear whether our findings can be generalized for various scanners other than the scanner used in this study. Finally, this study was an in vitro study using dental arch model. Further clinical studies are necessary to clarify how the scanning origin can play a role in data accuracy of RPD abutment teeth in digital impressions acquired by IOS in clinical situations.

## 5. Conclusions

Within the limitations of this study, it can be concluded that scanning origin has an impact on data accuracy of RPD abutment teeth region in digital impression acquired by an IOS, and scanning direction as well. To acquire superior data accuracy, it would be recommended to locate the scanning origin at end of remaining dental arch but not at remaining tooth isolated from other remaining teeth via mucosal area. Additionally, it was suggested that the scanning strategies directed from scanning origin directly to mucosal area would not be recommended.

## Figures and Tables

**Table 1:** *p*-values acquired by two-way ANOVA for T_G_, T_L_, and linear accuracy.

Variable	T_G_	T_L_	Linear Accuracy
Scanning strategy	<0.001 *	0.032 *	<0.001 *
Target abutment tooth	<0.001 *	<0.001 *	<0.001 *

*: statistically significant.

## Data Availability

The data presented in this study are available on reasonable request from the corresponding author.
